# Mapping outdoor habitat and abnormally small newborns to develop an ambient health hazard index

**DOI:** 10.1186/s12942-017-0117-5

**Published:** 2017-11-28

**Authors:** Charlene C. Nielsen, Carl G. Amrhein, Alvaro R. Osornio-Vargas, the DoMiNO Team

**Affiliations:** 1 Department of Earth and Atmospheric Sciences, University of Alberta, Edmonton, Canada; 2 Department of Pediatrics, University of Alberta, 3-591 ECHA, 11,405 87th Avenue, Edmonton, AB T6G 1C9 Canada

**Keywords:** Small for gestational age, Low birth weight at term, Pollution, GIS, Index

## Abstract

**Background:**

The geography of where pregnant mothers live is important for understanding outdoor environmental habitat that may result in adverse birth outcomes. We investigated whether more babies were born small for gestational age or low birth weight at term to mothers living in environments with a higher accumulation of outdoor hazards.

**Methods:**

Live singleton births from the Alberta Perinatal Health Program, 2006–2012, were classified according to birth outcome, and used in a double kernel density estimation to determine ratios of each outcome per total births. Individual and overlay indices of spatial models of 136 air emissions and 18 land variables were correlated with the small for gestational age and low birth weight at term, for the entire province and sub-provincially.

**Results:**

There were 24 air substances and land sources correlated with both small for gestational age and low birth weight at term density ratios. On the provincial scale, there were 13 air substances and 2 land factors; sub-provincial analysis found 8 additional air substances and 1 land source.

**Conclusion:**

This study used a combination of multiple outdoor variables over a large geographic area in an objective model, which may be repeated over time or in other study areas. The air substance-weighted index best identified where mothers having abnormally small newborns lived within areas of potential outdoor hazards. However, individual air substances and the weighted index provide complementary information.

## Background

**Fig. 1:**
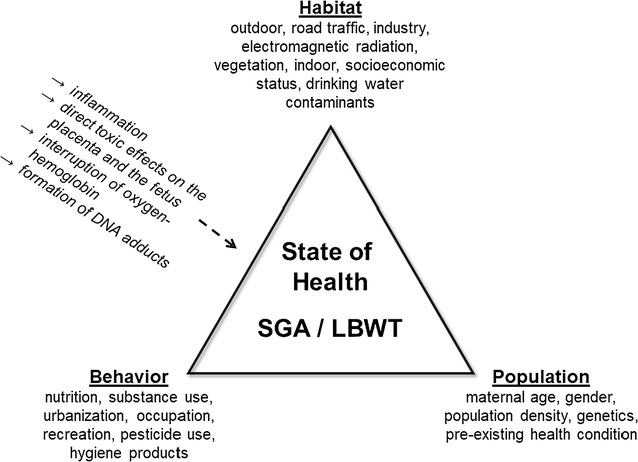
Meade’s triangle of human ecology for maternal exposures and small for gestational age (SGA) and low birth weight at term (LBWT): dashed arrow indicates hypothesized mechanisms

A truly ecologically-based study of health integrates habitat, population, and behavior—encompassing a more complete geography as framed by Meade’s triangle of human ecology [1, 2]. Three vertices conceptualize what is known about an important pediatric topic: maternal exposure to outdoor pollution and neonatal outcomes (Fig. 1). Here we focus on the lesser studied habitat vertex, specifically the outdoor environment, since less attention is traditionally given to incorporating ecological factors in understanding disease [1]. The location aspect of habitat—where pregnant women live, where industry and services are situated, where demographic groups congregate—is important because where one lives and where one starts out in life, even during fetal development, ultimately influences lifelong health [3–6].

Toxicant exposures and environmental influences on mothers during crucial stages of pregnancy may result in newborns that are too small or born too early. Adverse birth outcomes (ABO) are important markers of infant survival, development and future health. Our research focuses on being born too small, clinically defined as Small for Gestational Age (SGA) when newborns are below the 10th percentile weight based on sex and weeks of pregnancy, or Low Birth Weight at Term (LBWT) when newborns are less than 2500 g weight at term, 37 or more weeks gestation [7].

The province of Alberta, Canada, had a population of 3,645,257 at the 2011 Census [8]. That was a 10.8% increase from 2006 while the national average increase was 5.9%. For a land area of 640,082 km^2^, the population density was 5.7 persons/km^2^, where 83% of the population lived in or near urban centers. Alberta’s economic activities were focused on agriculture, natural resources, and nonrenewable energy—having a higher number of industrial facilities reporting to the National Pollutant Release Inventory (NPRI) than any other province or territory [9]. The NPRI is a valuable data source on industrial-based pollutants [10]. Alberta also has higher ABOs: SGA was 8.8% (Canada was 8.4%); and low birth weight for all gestational ages was 6.7% (Canada was 6.0%) [11]. Alberta rates also increased during 2000–2014: SGA from 10 to 11.5%; and low birth weight for all gestational ages from 6.1 to 6.7% [12].

ABO complications include death, physical and cognitive disabilities, and chronic health problems later in life, costing emotional stress and the majority of the health care expenses among all newborns [13]. Disorders related to short gestation and low birth weight are consistently ranked as the 2nd leading cause of infant death (congenital deformation is the leading cause) [14]—and have increased in Canada since the year 2000 [11, 12].

Abnormally small newborns are the result of growth restriction, which may be due to environmental pollutants thought to cause inflammation in mothers, direct toxic effects on the placenta and the fetus, interruption of oxygen-hemoglobin, or DNA damage represented by the formation of DNA adducts [15, 16].

The environment includes social, built, and natural features. Individual risks are also very important to ABOs, but are neither readily available nor easily mapped. These include personal, behavioral, social, and indoor exposures, such as: adequate prenatal care; food type and contaminants; rest, stress, and pre-existing health conditions; occupation and socioeconomic status; smoking and other substance use; drinking water contaminants. Our focus is on the outdoor environmental habitat because it is a common source of shared exposures susceptible to regulation (biology and behavior are not). These include air, water, human-constructed, and natural outdoor hazards, such as: industrial emissions; traffic pollution; agricultural chemical inputs of pesticides, herbicides, and fertilizers; electromagnetic radiation; proximity to oil and gas extraction activities; wildfire smoke.

Environmental health research has found many environmental factors to be associated with various health outcomes [17–25]. However, these are typically explored singly: one exposure or category of exposure at a time. A unified environmental measure may be constructed across multiple variables to encompass the complex nature of the overall environment.

Environmental indices have history: Inhaber had proposed an integrated national index for Canada in the 1970s [26]. Rather than relying on individual pollutants to reflect the state-of-the-environment, Inhaber mathematically combined such indicators for the purpose of resource allocation, ranking of locations, enforcement of standards, trend analysis, public information, or scientific research [27]. Under that premise, Messer et al. [28] developed a California county-level environmental quality index using principal components analysis (PCA) to calculate 5 environmental domains (air, water, land, built, and sociodemographic), which were then combined into a single index using PCA on the first components, and stratified by rural–urban continuum codes. Similarly, Messer et al.’s CalEnviroScreen 2.0 [29, 30] superimposed 19 individual indicators that related to pollution exposures, environmental conditions, and population characteristics, weighted and summed each set of indicators, and then multiplied together pollution and population (i.e. Threat × Vulnerability = Risk). We have not found similar environmental health indices available for Canada, or the province of Alberta, and especially none focused primarily on maternal exposures associated with ABO.

Using a Geographical Information System (GIS), we developed a simplified and reproducible index for Alberta by estimating and aggregating pollutants from communal outdoor factors. GIS supports the inclusion of diverse data and enables modelling of hazard-exposure-dose–response processes in space [31, 32]. To capture the relevant pollutant estimates, spatially and temporally appropriate GIS data files were overlaid to develop a vulnerability map of combined disparate (in theme and measurement units) environmental factors, similar to Messer et al. [28, 29]. The index will aid our examination of maternal ambient health hazards and abnormally small newborns by providing a relative ranking of locations across the province that are not limited by administrative boundaries.

Our research is part of the Data Mining and Neonatal Outcomes (DoMiNO) project that is exploring the collocation of adverse birth outcomes and environmental variables in Canada [33]. For our geographical perspective on the project we hypothesized that SGA or LBWT babies are more likely to be born to mothers living in environments with a higher number of outdoor hazards (especially pollutants) than in relatively healthier habitats with fewer exposure hazards. Our objective was to examine how the separate and combined exposures to the outdoor built-up, natural, and social environments of pregnant mothers coincided with patterns in adverse birth outcomes (ABO). We also expected that the large Alberta province would have regional variations in the outdoor environment and investigated this effect on the associations.

## Methods

### GIS parameters

We used Esri’s ArcGIS Desktop 10.5 software to perform all spatial database processing, management, distribution analyses, hazard estimations, and index calculations [34]. Proximity was extremely important in our spatial analysis; therefore, we customized an Alberta-focused map projection, based on the following parameters: name Azimuthal Equidistant; central meridian − 113.5; latitude of origin 53.5; linear unit meter (1.0); and geographic coordinate system (GCS) datum North American 1983 (NAD 1983). We projected all GIS data to this distance-preserving spatial reference.

For raster files we used a 250 m by 250 m cell size to reasonably represent both urban and rural areas in the very large study area, and to match the coarsest dataset: MODIS Terra satellite [35].

Because Alberta is landlocked, we included data features within 50 km surrounding the provincial boundary where available: by doing so, any potential pollutant source close to the outer edge of the province was included.

### Regional attribution

We produced sub-provincial maps of the percent ratios for each ABO to facilitate comparisons more meaningful to health care and environmental management. We assigned administrative attributes to postal code locations. This allowed grouping by health region [36] or airshed zone [37] because both are health-related administrative boundaries that help identify where there may be different outdoor factors of importance.

Health regions are designated by the provincial Ministry of Health to identify geographic areas where hospital boards or regional health authorities administer and deliver public health care, and are subject to change [38]. At the start of our study period, there were 9 health regions for Alberta (Table 2): Chinook Regional Health Authority (4821); Palliser Health Region (4822); Calgary Health Region (4823); David Thompson Regional Health Authority (4824); East Central Health (4825); Capital Health (4826); Aspen Regional Health Authority (4827); Peace Country Health (4828); and Northern Lights Health Region (4829).

Airshed zones are endorsed by the multi-stakeholder Clean Air Strategic Alliance (CASA) to identify geographic areas where the air quality is similar in emission sources, volumes, impacts, dispersion and administrative characteristics [39]. Because Alberta has several unique topographical, meteorological, or ecological conditions for resolving air quality, there are 9 airsheds currently recognized (Table 2): Alberta Capital Airshed Alliance (ACAA); Calgary Region Airshed Zone (CRAZ); Fort Air Partnership (FAP); Lakeland Industry and Community Association (LICA); Palliser Airshed Society (PAS); Parkland Airshed Management Zone (PAMZ); Peace Airshed Zone Association (PASZA); West Central Airshed Society (WCAS); and Wood Buffalo Environmental Association (WBEA). It is important to note that the entire province is not monitored by airshed zones, with the southwest corner, east-central, and majority of the north having no airshed (NA).

### Dependent variables

The Alberta Perinatal Health Program (APHP) provided anonymized data for the province of Alberta, from 2006 to 2012 [40]. We obtained ethics approval from the Research Ethics Board at the University of Alberta and the APHP.

We selected for live single births between 22 and 42 weeks gestation, and geocoded them to the centroid of the 6-character postal code of the mothers’ residences at the time of the birth registration. DMTI Spatial’s Platinum Postal Code Suite [41] provided the longitude and latitude coordinates for the years 2001–2013, which we uniquely selected to guarantee static locations through the entire study period. 95% of the original data had valid coordinates for use in spatial analyses. Using the previous definitions, we classified the birth records as binary variables identifying SGA or LBWT. Details are available in Serrano et al. [42].

To eliminate the confines of arbitrary administrative boundaries, we followed the double kernel density (DKD) method [43–48] to calculate distributions of SGA and LBWT, normalized by all births. DKD involves kernel density estimation—a non-parametric method that spreads point values across a surface by calculating the magnitude-per-unit area from points (representing the counts of birth events), fitted to a smoothly tapered function that spreads the values within a specified distance (25 km for this study) around each point [49]. Points within the radius that are further from the center are weighted lower than those closer, and helps indicate “hot spots”. Dividing each ABO by the kernel density of total births yielded ratios of the birth outcome that also masked locations of the residences, helping protect privacy.

### Independent variables

Personal maternal monitoring data were not available for this retrospective study. We used landscape features as spatial proxies of exposure hazards, as done in previously published research [32]. In total, we chose 18 outdoor sources, identified in published studies [17–23] or added for novel exploration (10 built; 5 social; 3 natural) plus 136 industrial air substance emissions. Table 1 lists the environmental variables and indicates specific characteristics and processing details.

**Table 1:** Outdoor environmental factors mapped for association with adverse birth outcomes

Category	Variable	Year	Feature	Method	Radius (km)	Units	Source
Built	136 air substances	Average 2006–2012	Point	Kernel density	10	tonnes or kg/km^2^	EnvCan [9]
	Industrial facilities	Unique 2006–2012	Point		3	#/km^2^	
	Roads	2012	Line			km/km^2^	StatsCan [69]
	Electrical power lines	2012	Line			km/km^2^	AltaLIS [70]
	Gas stations	2015	Point			#/km^2^	DMTI Spatial [41]
	Waste/landfills	2015	Point			#/km^2^	
	Oil/gas well pads	2012	Point			#/km^2^	ABMI [71]
	High density livestock operations	2012	Area	Focal statistics		#/km^2^	
	Mine sites	2012	Area			km^2^/km^2^	
	Cultivated lands	2012	Area			km^2^/km^2^	
	Nighttime lights	Average 2006–2012	Raster	None	0	index	NOAA [72]
Social	Food stores	2015	Point	Kernel density	3	#/km^2^	DMTI Spatial [41]
	Health care	2015	Point			#/km^2^	
	Hospitals	2015	Point			#/km^2^	
	Aboriginal lands	2016	Area	Focal statistics		km^2^/km^2^	NRCan [73]
	Neighborhood socioeconomic index	2006	Raster	None	0	index	Chan [74]
Natural	Vegetation/naturalness	Maximum 2006–2012	Raster	Focal statistics	3	index/km^2^	NASA [35]
	Water	2013	Area			km^2^/km^2^	NRCan [73]
	Wildfires	Average 2006–2012	Area		50	km^2^/km^2^	AgFor [75]

The time (year), distance threshold (radius in meters), units, and source are indicated for each

We applied kernel density to spread industrial emissions from the NPRI database as tonnes per area within a 10 km radius (based on distances determined from the project’s data mining algorithm [33]). We used the count of other point features—industrial facilities, gas stations, waste/landfills, oil/gas well pads, food stores, and health care/hospitals—in kernel density to calculate the number per area within a 3-km radius. We also applied kernel density to roads and electrical power lines to calculate length per area within a 3-km radius. A main advantage of using kernel density is it accounts for distance decay (features have less influence further away). When linear features are the input it also helps to approximate the number of intersections—important when analyzing pollution sources from roads because vehicles idle at intersections.

For areal features, we used focal statistics, also known as moving-window or neighborhood analyses, on binary surfaces of feedlots, mine sites, cultivated lands, aboriginal lands, water/blue space, and wildfires. The mean statistic on binary values of 1, indicating presence of the feature, and 0, indicating absence, yielded proportions. For vegetation/naturalness, the mean statistic returned the mean Normalized Difference Vegetation Index (NDVI), where higher values identify more chlorophyll-producing healthy green vegetation captured by the satellite imagery pixels. Except for the 50-km wildfire radius, all others had a 3-km radius. We accepted the original values for the coarser resolution nighttime lights and area-based, neighborhood-level socioeconomic index.

### Spearman’s rank correlation

We joined values from the DKD distributions and each independent variable surface extracted to unique postal codes where births occurred. Our data were non-normally distributed due to many zero values in both the dependent and independent variables. We used Python 2.7 software [50] with the pandas 0.16 site package [51] to calculate Spearman’s rank correlations among ABO and each environmental variable. To test the association of the combined environmental factors, we calculated a second set of Spearman’s rank correlations using DKD values to test the indices. Correlation was calculated for the entire province and aggregated by sub-provincial unit.

### Overlay analysis

Overlay analysis is a simple and reproducible method to combine several inputs into a single output [52]. It is most common for optimal site selection and suitability modeling, especially for mapping habitat. The class values represent rankings from higher to lower suitability or risk. In our study, we applied it to essentially map “reverse suitability” to identify maternal ambient health hazards.

Because the values of continuous surfaces varied in measurement units, we standardized them into a similar ratio scale by reclassifying the environmental variables into five standard classes using quintiles. The ordering of the reclassification corresponded with the direction of the correlation: most were straightforward but if the variable was negatively correlated then the reclassification was applied in a backwards fashion; e.g. vegetation, water, and socioeconomic status classes were ranked 5–1 because lower original values were considered to be more hazardous. We calculated the sub-indices as weighted sum overlays with equal weightings on air substances and land-based sources separately, which were then overlaid together. We were interested in preserving the combined effects of the industrial air substances; therefore, in addition to an equal weighted sum of both, we also approximated a conservative two-thirds (0.7) weighting to the air substances summed with a one-third (0.3) weighting of the land-based sources. In the two different indices—Overlay Equal and Overlay 0.7/0.3—the class rankings were accumulations that represent where the study area had more environmental hazards.

**Fig. 2:**
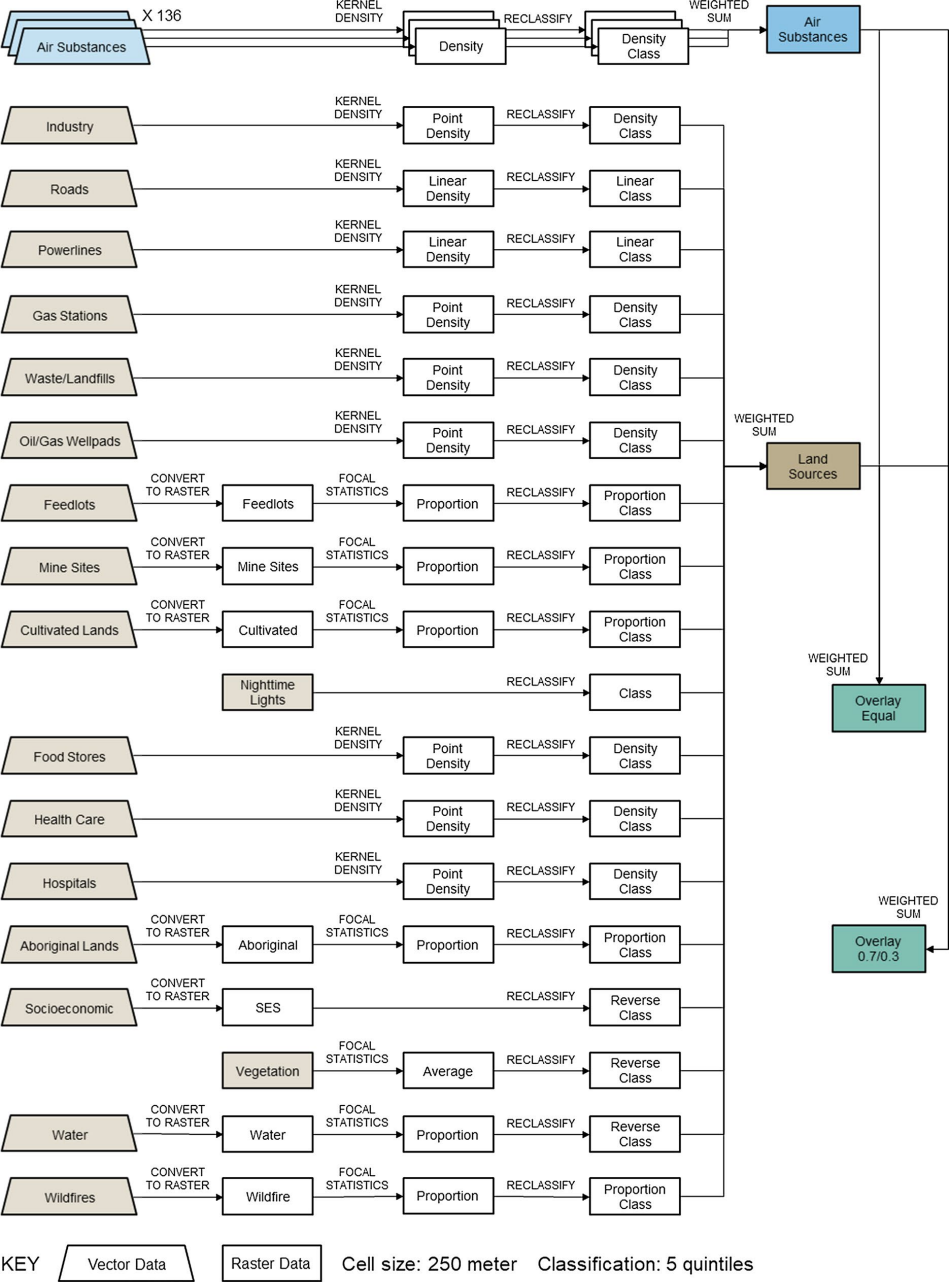
Flow chart of standard GIS commands for constructing the indices (colored boxes); details on parameter values for each of the independent variables are specified in Table 1

Overall, the reverse suitability indices were calculated by modeling each individual pollutant surface using distance-centered analyses (i.e. kernel densities and focal statistics), reclassified into quintiles of class rankings, and overlaid as weighted sums. The detailed GIS methods for the map-based calculations of all the independent variables and subsequent indices are specified in Table 1 (i.e. features, methods, and radii) and shown graphically in Fig. 2.

## Results

### Spatial distribution of adverse birth outcomes

Table 2 shows raw counts of births, SGA, and LBWT, based on valid postal codes. For 2006–2012, the entire province of Alberta had 333,247 births with a valid spatial location (95% of total registered), allocated to 53,399 postal codes. 29,679 geocoded births were classified as SGA (8.9%) and 5485 were classified as LBWT at term (1.6%).

**Table 2:** Alberta’s sub-provincial units and descriptive statistics, in descending order of birth number

Unit	Map code	Name	Area (km^2^)	Postal codes	Geolocated births	SGA	LBWT
Province	none	Alberta	663,563	53,399	333,247	29,679	5485
Health Region	4823	Calgary Health Region	39,350	20,537	121,965	12,543	2339
	4826	Capital Health	11,883	20,004	99,691	8596	1566
	4824	David Thompson Regional Health Authority	61,578	3325	29,766	2394	476
	4827	Aspen Regional Health Authority	137,639	1440	18,004	1252	222
	4821	Chinook Regional Health Authority	26,062	2406	16,639	1342	233
	4828	Peace Country Health	123,870	1580	16,428	1188	215
	4829	Northern Lights Health Region	189,696	1073	11,097	808	147
	4822	Palliser Health Region	39,772	1723	9920	858	147
	4825	East Central Health	33,812	1311	9737	698	140
Airshed Zone	CRAZ	Calgary Regional Airshed Zone	32,372	20,530	120,392	12,409	2310
	ACAA	Alberta Capital Airshed Alliance	4933	19,474	95,085	8284	1503
	NA	No Airshed Zone	362,439	4867	47,527	3509	647
	PAMZ	Parkland Airshed Management Zone	40,936	2774	24,896	1978	387
	PASZA	Peace Airshed Zone Association	45,892	1409	12,475	927	175
	PAS	Palliser Airshed Society	39,900	1723	9920	858	147
	WBEA	Wood Buffalo Environmental Association	69,214	1061	7540	627	115
	WCAS	West Central Airshed Society	47,142	612	7386	559	107
	LICA	Lakeland Industrial Community Association	16,215	455	4479	293	43
	FAP	Fort Air Partnership	4519	494	3547	235	51

**Fig. 3:**
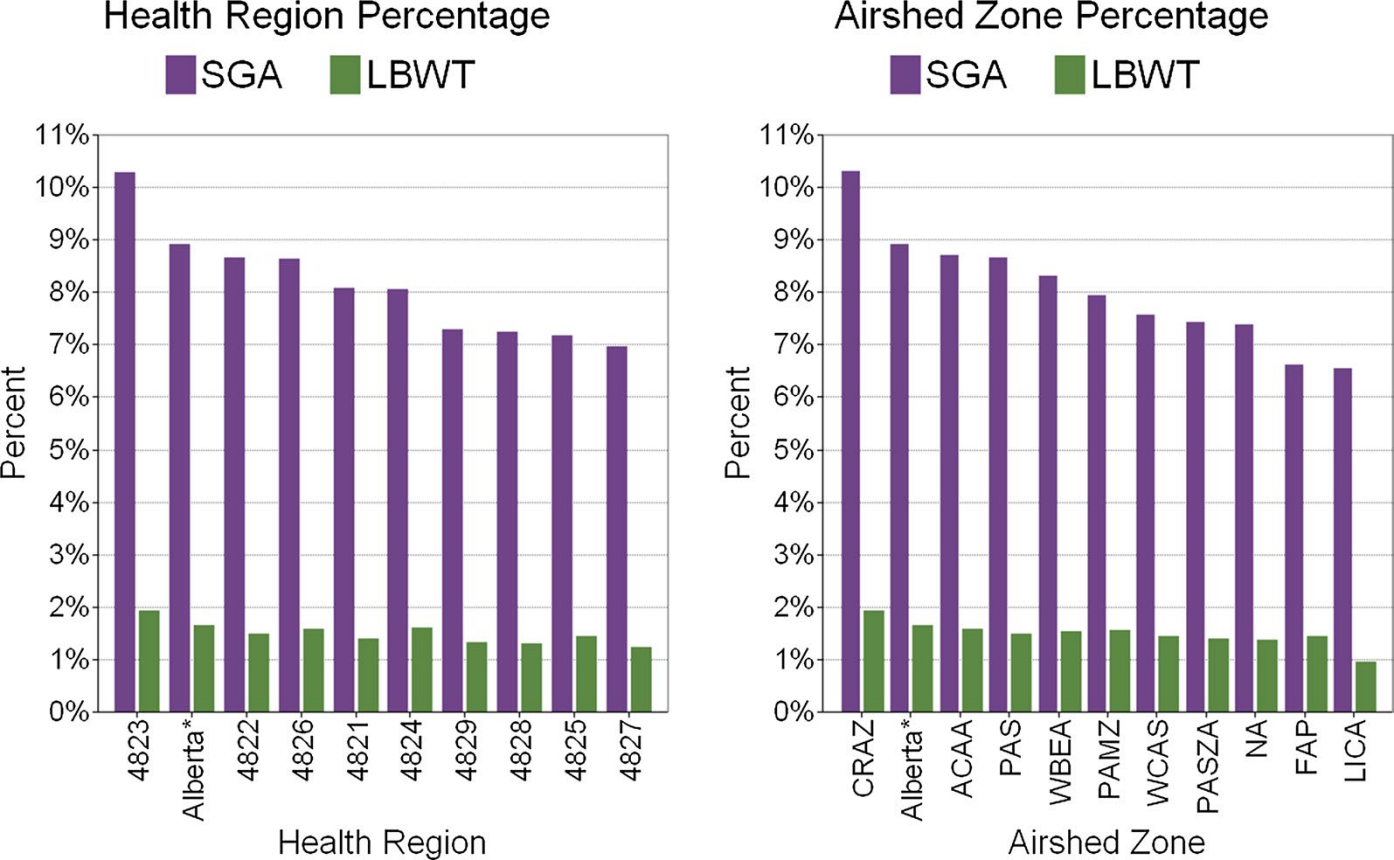
Percentages of births having small for gestational age (SGA) or low birth weight at term (LBWT) in sub-provincial units (* indicates value for the whole province)

Figure 3 depicts the percentages of ABO for each sub-provincial unit relative to Alberta (marked by *). For health regions, SGA ranged from 7.0 to 10.3% and LBWT ranged from 1.2 to 1.9%. Health region 4823 had the highest number of births (n = 121,965), highest SGA (n = 12,543, 10.3%), and highest LBWT (n = 2339, 1.9%); 4825 had the lowest number of births (n = 9737), SGA (n = 698, 7.2%), and LBWT (n = 140, 1.4%); but 4827 had the lowest SGA (n = 1252, 7.0%) and LBWT (n = 222, 1.2%). For airshed zones, SGA ranged from 6.5 to 10.3% and LBWT ranged from 1.0 to 1.9%. Airshed zone CRAZ had the highest number of births (n = 120,392), SGA (n = 12,409, 10.3%), and LBWT (n = 2310, 1.9%); FAP had the lowest number of births (n = 3.547) and LICA had the lowest SGA (n = 293, 6.5%) and lowest LBWT (n = 43, 1.0%).

**Fig. 4:**
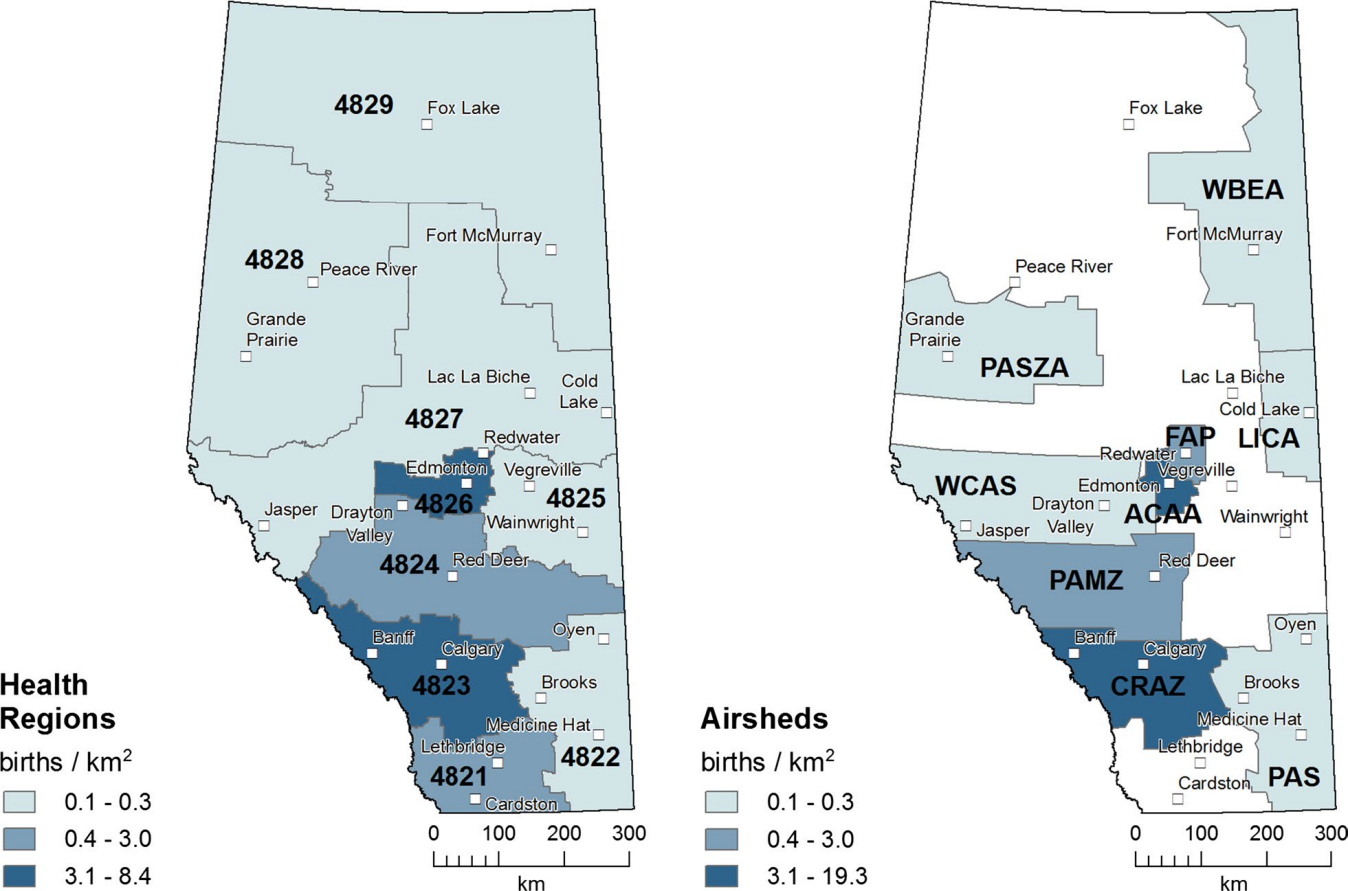
Births per area ratios in sub-provincial units

The distributions of births per area in Fig. 4 show higher concentrations of more than 3 births per km^2^ in the sub-provincial units containing the major cities of Edmonton and Calgary, with medium densities in the adjacent units and in the airshed zones containing Grande Prairie (west-central) and Cold Lake (east-central).

**Fig. 5:**
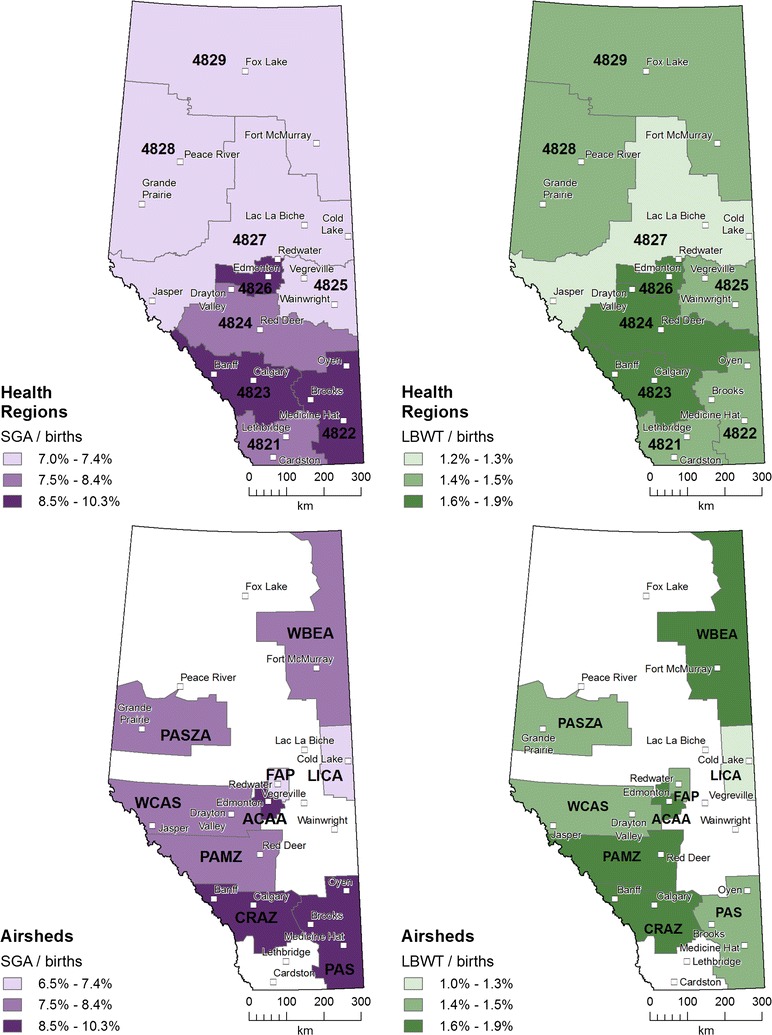
Adverse birth outcome (ABO) ratios in sub-provincial units

The patterns differ by sub-provincial unit for ABOs mapped as numbers per births (Fig. 5). SGA is highest in the units containing Edmonton and along the west–east Banff–Calgary-Brooks corridor. Health regions have medium SGA adjacent to the high SGA. Airsheds also show medium SGA in the west and north-east. LBWT is highest in the north–south Edmonton-Red Deer-Calgary corridor. Medium LBWT is adjacent to the higher units, except for the northern health regions containing Grande Prairie-Peace River and Fort McMurray-Fox Lake. The lower LBWT in the central health region 4827 separates the province; LBWT in the airshed containing Cold Lake is the lowest in the province.

**Fig. 6:**
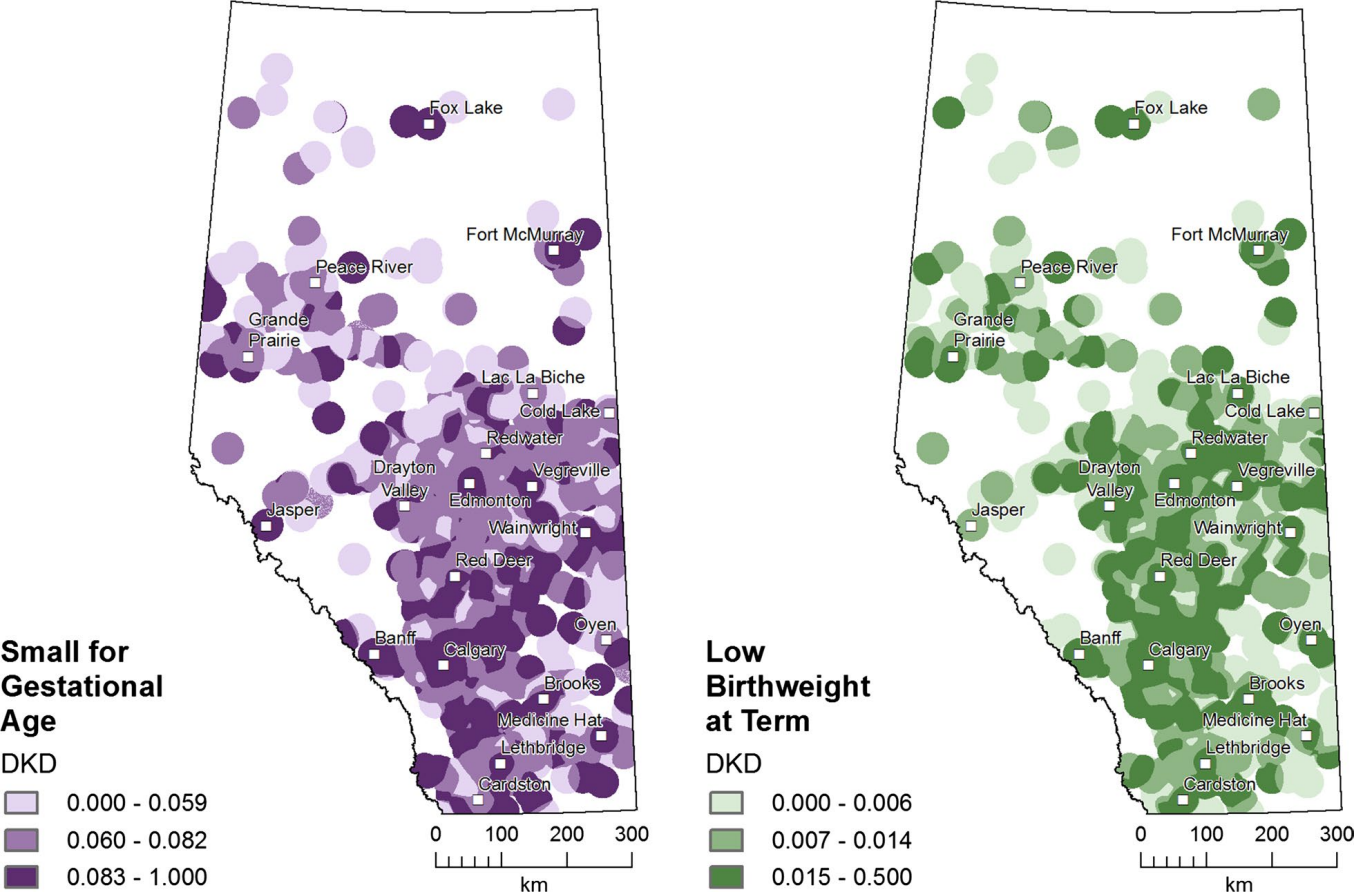
Double kernel density (DKD) distributions of small for gestational age (SGA) and low birth weight at term (LBWT) are ratios of the adverse birth outcome (ABO) per area divided by total births per area, each within a 25 km radius; DKD is dimensionless

Figure 6 maps the results of the DKD method for each ABO. Both ABOs cover the same areas of the province and the darker colors indicate higher values for SGA (purple) and LBWT (green). The result of DKD is a continuous value, but the maps classified with tertiles visually enhance the slightly different distributions for SGA and LBWT: urban (Edmonton and Calgary) areas shared highest values for both ABO; central areas had more LBWT; and southeast areas had more SGA.

### Hazard mapping

The Spearman’s rank correlation values were sorted in descending order for each of the independent variables (Table 3). Provincially, variables having correlations greater than 0.40 (low value accepted since data were not adjusted for epidemiological factors because they were not available for mapping) with SGA were: i-Butyl alcohol (*rho* = 0.56); Asbestos; Nighttime Light; Toluenediisocyanate; Toluene-2,4-diisocyanate; Toluene-2,6-diisocyanate; Chromium Aluminum; Hydrogen sulphide; Road; 2-Ethoxyethanol; *Nickel; Quinoline; Aniline; Cyclohexane; Acetaldehyde; and *Phosphorus (*rho* = 0.42). Variables with correlations greater than 0.40 with LBWT were: i-Butyl alcohol (*rho* = 0.54); Asbestos; Toluenediisocyanate; Toluene-2,4-diisocyanate; Toluene-2,6-diisocyanate; Aluminum; Chromium; Nighttime Light; Hydrogen sulphide; 2-Ethoxyethanol; Quinoline; Aniline; Road; Cyclohexane; Acetaldehyde; *Isopropyl alcohol; and *Ethylene oxide (*rho* = 0.41). Both ABOs were strongly associated with 15 air substances (the asterisk * marks those that differed: Nickel and Phosphorous for SGA; Ethylene oxide and Isopropyl alcohol for LBWT) and 2 land sources (both Nighttime Light and Road). Both ABOs had negative correlations (< − 0.40) with Vegetation (SGA *rho* = − 0.56; LBWT *rho* = − 0.48), Oil/Gas Wellpad (SGA *rho* = − 0.53; LBWT *rho* = − 0.49), and Cultivated Land (SGA *rho* = − 0.47; LBWT *rho* = − 0.41).

**Table 3:** Spearman’s rank correlations of small for gestational age (SGA) and low birth weight at term (LBWT) with air substances and land sources (*), in descending correlation *rho* values

Variable	Province *rho*		Health Region count (*rho* range)		Airshed Zone count (*rho* range)	
	SGA	LBWT	SGA	LBWT	SGA	LBWT
i-Butyl alcohol	**0.56**	**0.54**	1 (0.02 to 0.81)	3 (0.42 to 0.80)	1 (0.32 to 0.81)	2 (0.45 to 0.80)
Asbestos (friable form)	**0.54**	**0.52**	1 (0.73 to 0.73)	1 (0.67 to 0.67)	1 (0.73 to 0.73)	1 (0.67 to 0.67)
*Nighttime Light	**0.51**	**0.47**	2 (− 0.17 to 0.48)	1 (− 0.50 to 0.42)	2 (− 0.40 to 0.51)	3 (− 0.50 to 0.52)
Toluenediisocyanate (mixed isomers)	**0.49**	**0.51**	0 (0.26 to 0.26)	1 (0.42 to 0.42)	0 (0.26 to 0.26)	1 (0.42 to 0.42)
Toluene-2,4-diisocyanate	**0.49**	**0.50**	0 (0.26 to 0.26)	1 (0.41 to 0.41)	0 (0.26 to 0.26)	1 (0.41 to 0.41)
Toluene-2,6-diisocyanate	**0.49**	**0.50**	0 (0.26 to 0.26)	1 (0.41 to 0.41)	0 (0.26 to 0.26)	1 (0.41 to 0.41)
Chromium (and its compounds)	**0.48**	**0.47**	2 (− 0.07 to 0.68)	2 (− 0.07 to 0.77)	2 (− 0.23 to 0.68)	2 (− 0.26 to 0.77)
Aluminum (fume or dust)	**0.48**	**0.49**	1 (0.20 to 0.67)	1 (0.25 to 0.76)	1 (− 0.18 to 0.67)	1 (− 0.20 to 0.76)
Hydrogen sulphide	**0.47**	**0.47**	**4 (**− **0.09 to 0.59)**	3 (− 0.06 to 0.67)	3 (− 0.34 to 0.59)	3 (− 0.35 to 0.67)
*Road	**0.46**	**0.42**	2 (− 0.11 to 0.47)	0 (− 0.37 to 0.38)	3 (− 0.01 to 0.60)	2 (− 0.37 to 0.51)
2-Ethoxyethanol	**0.44**	**0.46**	0 (0.25 to 0.25)	1 (0.41 to 0.41)	0 (0.25 to 0.25)	1 (0.41 to 0.41)
Nickel (and its compounds)	**0.44**	**0.39**	2 (− 0.20 to 0.66)	3 (− 0.88 to 0.75)	2 (− 0.24 to 0.66)	2 (− 0.88 to 0.75)
Quinoline (and its salts)	**0.43**	**0.46**	0 (0.25 to 0.25)	1 (0.40 to 0.40)	0 (0.25 to 0.25)	1 (0.40 to 0.40)
Aniline (and its salts)	**0.43**	**0.45**	0 (0.25 to 0.25)	1 (0.40 to 0.40)	0 (0.25 to 0.25)	1 (0.40 to 0.40)
Cyclohexane	**0.42**	**0.42**	3 (− 0.07 to 0.81)	3 (− 0.07 to 0.81)	3 (− 0.27 to 0.81)	3 (− 0.30 to 0.81)
Acetaldehyde	**0.42**	**0.42**	**4 (**− **0.41 to 0.60)**	3 (− 0.07 to 0.59)	**4 (**− **0.34 to 0.60)**	3 (− 0.34 to 0.59)
Phosphorus (total)	**0.42**	0.38	1 (− 0.20 to 0.49)	1 (− 0.88 to 0.44)	1 (− 0.49 to 0.52)	1 (− 0.88 to 0.46)
Isopropyl alcohol	0.40	**0.41**	2 (− 0.40 to 0.52)	3 (− 0.47 to 0.53)	2 (− 0.60 to 0.77)	2 (− 0.67 to 0.52)
PAHs, Total unspeciated	0.40	0.40	0 (0.30 to 0.32)	1 (0.33 to 0.43)	0 (0.26 to 0.32)	1 (0.29 to 0.43)
Ethylene oxide	0.36	**0.41**	0 (− 0.43 to 0.25)	1 (− 0.20 to 0.41)	0 (− 0.30 to 0.25)	1 (− 0.32 to 0.41)
Ammonia (total)	0.36	0.34	1 (− 0.41 to 0.63)	1 (− 0.75 to 0.73)	1 (− 0.50 to 0.53)	1 (− 0.75 to 0.66)
Phosphorus (yellow or white)	0.35	0.37	0 (− 0.14 to 0.05)	0 (− 0.14 to 0.23)	0 (− 0.15 to 0.05)	0 (− 0.14 to 0.23)
Methylenebis(phenylisocyanate)	0.34	0.38	0 (− 0.43 to 0.23)	0 (− 0.49 to 0.38)	0 (0.13 to 0.23)	1 (0.38 to 0.72)
PM_10_—Particulate Matter <= 10 Microns	0.33	0.30	3 (− 0.33 to 0.89)	3 (− 0.83 to 0.62)	3 (− 0.75 to 0.93)	**4 (**− **0.83 to 0.68)**
n-Butyl alcohol	0.31	0.32	1 (− 0.71 to 0.79)	1 (− 0.75 to 0.81)	1 (0.19 to 0.79)	1 (0.36 to 0.81)
Dichloromethane	0.31	0.31	1 (0.27 to 0.72)	2 (0.42 to 0.74)	1 (− 0.22 to 0.71)	2 (− 0.25 to 0.73)
Ethylene	0.30	0.33	3 (0.02 to 0.80)	3 (0.02 to 0.79)	3 (− 0.32 to 0.80)	3 (− 0.33 to 0.79)
Styrene	0.30	0.31	1 (0.00 to 0.81)	1 (− 0.01 to 0.82)	1 (− 0.32 to 0.83)	1 (− 0.32 to 0.85)
Lead (and its compounds)	0.30	0.30	3 (− 0.07 to 0.68)	**4 (**− **0.26 to 0.76)**	3 (− 0.23 to 0.87)	3 (− 0.65 to 0.76)
*Food Store	0.28	0.28	1 (− 0.18 to 0.58)	1 (− 0.23 to 0.57)	2 (− 0.18 to 0.58)	1 (− 0.17 to 0.57)
Cumene	0.27	0.27	1 (0.29 to 0.58)	2 (0.44 to 0.61)	1 (− 0.14 to 0.57)	2 (− 0.09 to 0.60)
Methyl isobutyl ketone	0.25	0.26	1 (0.07 to 0.69)	1 (0.25 to 0.73)	1 (0.07 to 0.67)	1 (0.08 to 0.71)
Xylene (mixed isomers)	0.24	0.26	1 (− 0.71 to 0.54)	**5 (**− **0.74 to 0.59)**	2 (− 0.65 to 0.74)	**5 (**− **0.47 to 0.87)**
Sulphur dioxide	0.24	0.20	**5 (**− **0.27 to 0.88)**	3 (− 0.87 to 0.74)	**4 (**− **0.35 to 0.91)**	2 (− 0.87 to 0.68)
Manganese (and its compounds)	0.24	0.21	3 (− 0.03 to 0.68)	**4 (**− **0.34 to 0.72)**	3 (− 0.50 to 0.65)	3 (− 0.71 to 0.70)
Fluorene—PAH	0.23	0.13	2 (− 0.32 to 0.50)	2 (− 0.89 to 0.55)	2 (− 0.49 to 0.52)	2 (− 0.89 to 0.53)
2-Butoxyethanol	0.22	0.23	1 (− 0.69 to 0.66)	1 (− 0.70 to 0.66)	1 (0.11 to 0.64)	1 (0.11 to 0.64)
*Gas Station	0.20	0.20	1 (− 0.23 to 0.58)	0 (− 0.25 to 0.30)	2 (− 0.41 to 0.56)	1 (− 0.42 to 0.58)
Naphthalene	0.20	0.14	1 (− 0.21 to 0.59)	2 (− 0.89 to 0.63)	1 (− 0.40 to 0.61)	2 (− 0.89 to 0.65)
Propylene	0.18	0.19	1 (− 0.11 to 0.68)	1 (− 0.01 to 0.71)	1 (− 0.30 to 0.70)	1 (− 0.31 to 0.73)
*Health Care	0.17	0.17	2 (− 0.21 to 0.51)	0 (− 0.36 to 0.24)	2 (− 0.22 to 0.53)	0 (− 0.49 to 0.28)
Volatile Organic Compounds (VOCs)	0.17	0.15	3 (− 0.45 to 0.88)	2 (− 0.74 to 0.67)	2 (− 0.45 to 0.92)	1 (− 0.74 to 0.69)
Toluene	0.17	0.19	1 (− 0.05 to 0.68)	2 (− 0.35 to 0.72)	2 (− 0.66 to 0.74)	3 (− 0.46 to 0.89)
Formic acid	0.14	0.15	0 (− 0.50 to 0.39)	1 (− 0.50 to 0.52)	0 (− 0.49 to 0.39)	1 (− 0.50 to 0.52)
PM_2.5_—Particulate Matter <= 2.5 Microns	0.14	0.11	**4 (**− **0.57 to 0.88)**	3 (− 0.73 to 0.59)	3 (− 0.57 to 0.91)	**4 (**− **0.73 to 0.61)**
1,2,4-Trimethylbenzene	0.13	0.18	1 (− 0.05 to 0.57)	2 (− 0.07 to 0.60)	2 (− 0.38 to 0.67)	1 (− 0.47 to 0.60)
Formaldehyde	0.12	0.10	2 (− 0.41 to 0.70)	3 (− 0.81 to 0.71)	3 (− 0.29 to 0.83)	3 (− 0.81 to 0.70)
Vanadium (except when in an alloy) and its compounds	0.11	0.12	1 (− 0.05 to 0.49)	1 (0.05 to 0.54)	1 (− 0.21 to 0.47)	1 (− 0.16 to 0.52)
Carbon disulphide	0.10	0.10	1 (− 0.21 to 0.41)	1 (− 0.28 to 0.52)	1 (− 0.26 to 0.75)	0 (− 0.32 to 0.30)
Benzo(g,h,i)perylene—PAH	0.10	0.05	2 (− 0.27 to 0.60)	2 (− 0.89 to 0.65)	2 (− 0.49 to 0.59)	2 (− 0.89 to 0.64)
Indeno(1,2,3-c,d)pyrene—PAH	0.09	0.05	2 (− 0.27 to 0.61)	2 (− 0.89 to 0.65)	2 (− 0.49 to 0.59)	2 (− 0.89 to 0.64)
Pyrene—PAH	0.09	0.03	2 (− 0.27 to 0.62)	2 (− 0.89 to 0.66)	2 (− 0.49 to 0.61)	2 (− 0.89 to 0.65)
Perylene—PAH	0.09	0.04	2 (− 0.27 to 0.60)	2 (− 0.89 to 0.64)	2 (− 0.49 to 0.59)	2 (− 0.89 to 0.64)
Benzo(a)phenanthrene—PAH	0.09	0.04	2 (− 0.27 to 0.61)	2 (− 0.89 to 0.64)	2 (− 0.49 to 0.60)	2 (− 0.89 to 0.64)
Benzo(e)pyrene—PAH	0.08	0.04	2 (− 0.27 to 0.61)	2 (− 0.89 to 0.65)	2 (− 0.27 to 0.59)	2 (− 0.89 to 0.64)
Benzo(a)anthracene—PAH	0.08	0.04	2 (− 0.27 to 0.61)	2 (− 0.89 to 0.65)	2 (− 0.27 to 0.59)	2 (− 0.89 to 0.64)
Fluoranthene—PAH	0.08	0.02	2 (− 0.27 to 0.61)	2 (− 0.89 to 0.65)	2 (− 0.27 to 0.60)	2 (− 0.89 to 0.64)
Methyl ethyl ketone	0.08	0.08	1 (− 0.21 to 0.80)	1 (− 0.31 to 0.82)	1 (− 0.32 to 0.79)	1 (− 0.32 to 0.81)
Benzene	0.07	0.09	0 (− 0.12 to 0.29)	1 (− 0.08 to 0.54)	1 (− 0.66 to 0.70)	2 (− 0.31 to 0.88)
Benzo(k)fluoranthene—PAH	0.07	0.02	2 (− 0.27 to 0.60)	2 (− 0.89 to 0.65)	2 (− 0.49 to 0.59)	2 (− 0.89 to 0.64)
*Aboriginal Land	0.07	0.05	0 (− 0.35 to 0.00)	0 (− 0.29 to 0.03)	0 (− 0.64 to − 0.04)	0 (− 0.29 to 0.23)
Diethanolamine (and its salts)	0.07	0.06	1 (0.00 to 0.41)	1 (− 0.01 to 0.46)	1 (− 0.25 to 0.44)	1 (− 0.25 to 0.49)
Benzo(a)pyrene—PAH	0.06	0.02	2 (− 0.27 to 0.57)	2 (− 0.89 to 0.61)	2 (− 0.49 to 0.56)	2 (− 0.89 to 0.60)
Aluminum oxide (fibrous forms)	0.06	0.05	1 (0.81 to 0.81)	1 (0.80 to 0.80)	1 (0.81 to 0.81)	1 (0.81 to 0.81)
Benzo(j)fluoranthene—PAH	0.06	0.01	2 (− 0.27 to 0.57)	2 (− 0.89 to 0.61)	2 (− 0.49 to 0.56)	2 (− 0.89 to 0.60)
Benzo(b)fluoranthene—PAH	0.06	0.01	2 (− 0.27 to 0.57)	2 (− 0.89 to 0.61)	2 (− 0.49 to 0.56)	2 (− 0.89 to 0.60)
n-Hexane	0.04	0.05	2 (− 0.09 to 0.63)	2 (− 0.31 to 0.65)	2 (− 0.36 to 0.53)	2 (− 0.47 to 0.85)
Calcium fluoride	0.03	0.02	1 (0.67 to 0.67)	1 (0.71 to 0.71)	1 (0.08 to 0.67)	1 (0.08 to 0.70)
Carbonyl sulphide	0.03	0.03	1 (0.04 to 0.41)	2 (− 0.28 to 0.52)	2 (− 0.26 to 0.75)	1 (− 0.31 to 0.48)
*Mine site	0.02	0.00	2 (− 0.35 to 0.43)	**4 (**− **0.41 to 0.50)**	1 (− 0.21 to 0.53)	2 (− 0.60 to 0.57)
Biphenyl	0.01	0.00	1 (0.60 to 0.60)	1 (0.64 to 0.64)	1 (− 0.16 to 0.59)	1 (− 0.11 to 0.63)
*Waste/Landfill	0.01	0.05	0 (− 0.29 to 0.30)	1 (− 0.31 to 0.42)	1 (− 0.29 to 0.80)	1 (− 0.27 to 0.42)
Ethylene glycol	0.00	0.00	1 (− 0.69 to 0.41)	2 (− 0.85 to 0.53)	1 (− 0.44 to 0.75)	1 (− 0.85 to 0.49)
Hydrogen fluoride	0.00	0.00	1 (0.04 to 0.55)	1 (− 0.05 to 0.59)	1 (− 0.09 to 0.54)	1 (− 0.01 to 0.58)
Methyl tert-butyl ether	0.00	− 0.01	1 (0.77 to 0.77)	1 (0.78 to 0.78)	1 (0.15 to 0.76)	1 (0.15 to 0.77)
n,n-Dimethylformamide	0.00	− 0.02	1 (0.72 to 0.72)	1 (0.74 to 0.74)	1 (0.15 to 0.71)	1 (0.15 to 0.73)
Vinyl acetate	0.00	− 0.01	1 (0.56 to 0.56)	1 (0.58 to 0.58)	1 (0.54 to 0.54)	1 (0.57 to 0.57)
N-Methyl-2-pyrrolidone	0.00	− 0.01	1 (0.53 to 0.53)	1 (0.57 to 0.57)	1 (0.15 to 0.52)	1 (0.15 to 0.56)
Isoprene	0.00	0.00	0 (0.00 to 0.00)	0 (− 0.01 to − 0.01)	0 (− 0.01 to − 0.01)	0 (− 0.01 to − 0.01)
Titanium tetrachloride	0.00	0.00	0 (0.00 to 0.00)	0 (− 0.01 to − 0.01)	0 (− 0.01 to − 0.01)	0 (− 0.01 to − 0.01)
Methanol	− 0.01	− 0.01	1 (− 0.39 to 0.52)	1 (− 0.79 to 0.48)	0 (− 0.39 to 0.30)	1 (− 0.79 to 0.48)
Cresol (all isomers and their salts)	− 0.01	− 0.02	1 (0.00 to 0.55)	1 (− 0.32 to 0.59)	1 (− 0.49 to 0.54)	1 (− 0.72 to 0.58)
Carbon monoxide	− 0.01	− 0.04	2 (− 0.41 to 0.89)	1 (− 0.82 to 0.60)	2 (− 0.41 to 0.92)	2 (− 0.82 to 0.48)
Trichloroethylene	− 0.01	− 0.01	1 (0.49 to 0.49)	1 (0.53 to 0.53)	1 (− 0.24 to 0.51)	1 (− 0.27 to 0.56)
p-Phenylenediamine (and its salts)	− 0.02	− 0.01	0 (− 0.03 to − 0.03)	0 (− 0.03 to − 0.03)	0 (− 0.18 to − 0.18)	0 (− 0.13 to − 0.13)
Acrolein	− 0.02	− 0.10	1 (0.06 to 0.70)	1 (0.00 to 0.67)	1 (0.05 to 0.75)	0 (0.05 to 0.20)
Hexavalent chromium (and its compounds)	− 0.02	0.02	0 (− 0.07 to 0.31)	0 (− 0.32 to 0.17)	0 (− 0.50 to 0.08)	0 (− 0.71 to 0.25)
Dibenzo(a,i)pyrene—PAH	− 0.02	− 0.09	1 (− 0.21 to 0.54)	1 (− 0.89 to 0.59)	1 (− 0.21 to 0.53)	1 (− 0.89 to 0.58)
7H-Dibenzo(c,g)carbazole—PAH	− 0.02	− 0.11	1 (− 0.21 to 0.55)	1 (− 0.89 to 0.59)	1 (− 0.21 to 0.54)	1 (− 0.89 to 0.58)
tert-Butyl alcohol	− 0.02	− 0.03	0 (0.31 to 0.31)	0 (0.35 to 0.35)	0 (0.17 to 0.29)	0 (0.17 to 0.34)
Molybdenum trioxide	− 0.03	− 0.11	1 (− 0.20 to 0.44)	1 (− 0.88 to 0.49)	1 (− 0.20 to 0.43)	1 (− 0.88 to 0.48)
Acenaphthene—PAH	− 0.03	− 0.11	2 (− 0.21 to 0.63)	2 (− 0.89 to 0.66)	2 (− 0.49 to 0.61)	2 (− 0.89 to 0.66)
Chlorine	− 0.04	− 0.02	2 (− 0.55 to 0.54)	3 (− 0.32 to 0.57)	2 (− 0.55 to 0.56)	3 (− 0.72 to 0.60)
Phenanthrene—PAH	− 0.05	− 0.11	1 (− 0.27 to 0.49)	2 (− 0.89 to 0.44)	1 (− 0.49 to 0.52)	1 (− 0.89 to 0.46)
*Industrial facility	− 0.05	− 0.02	1 (− 0.18 to 0.43)	0 (− 0.46 to 0.29)	1 (− 0.62 to 0.47)	1 (− 0.59 to 0.40)
*Hospital	− 0.05	− 0.03	1 (− 0.19 to 0.44)	0 (− 0.30 to 0.19)	2 (− 0.33 to 0.70)	0 (− 0.47 to 0.19)
5-Methylchrysene—PAH	− 0.06	− 0.22	0 (− 0.21 to − 0.21)	0 (− 0.89 to − 0.89)	0 (− 0.21 to − 0.21)	0 (− 0.89 to − 0.89)
1-Nitropyrene—PAH	− 0.06	− 0.22	0 (− 0.21 to − 0.21)	0 (− 0.89 to − 0.89)	0 (− 0.21 to − 0.21)	0 (− 0.89 to − 0.89)
Dibenzo(a,e)fluoranthene—PAH	− 0.06	− 0.22	0 (− 0.21 to − 0.21)	0 (− 0.89 to − 0.89)	0 (− 0.21 to − 0.21)	0 (− 0.89 to − 0.89)
Dibenzo(a,h)pyrene—PAH	− 0.06	− 0.22	0 (− 0.21 to − 0.21)	0 (− 0.89 to − 0.89)	0 (− 0.21 to − 0.21)	0 (− 0.89 to − 0.89)
Dibenzo(a,l)pyrene—PAH	− 0.06	− 0.22	0 (− 0.21 to − 0.21)	0 (− 0.89 to − 0.89)	0 (− 0.21 to − 0.21)	0 (− 0.89 to − 0.89)
Dibenz(a,h)acridine—PAH	− 0.06	− 0.22	0 (− 0.21 to − 0.02)	0 (− 0.89 to − 0.01)	0 (− 0.21 to − 0.02)	0 (− 0.89 to − 0.01)
Dibenzo(a,e)pyrene—PAH	− 0.06	− 0.22	0 (− 0.21 to − 0.02)	0 (− 0.89 to − 0.01)	0 (− 0.21 to − 0.02)	0 (− 0.89 to − 0.01)
Anthracene	− 0.06	− 0.22	0 (− 0.21 to − 0.03)	0 (− 0.89 to − 0.03)	0 (− 0.21 to − 0.19)	0 (− 0.89 to − 0.14)
Dibenz(a,j)acridine—PAH	− 0.06	− 0.13	1 (− 0.21 to 0.55)	1 (− 0.89 to 0.59)	1 (− 0.21 to 0.54)	1 (− 0.89 to 0.58)
Sulphuric acid	− 0.06	− 0.10	2 (− 0.21 to 0.52)	2 (− 0.89 to 0.56)	2 (− 0.50 to 0.54)	2 (− 0.89 to 0.58)
Ethylbenzene	− 0.07	− 0.05	2 (− 0.71 to 0.46)	3 (− 0.74 to 0.55)	2 (− 0.37 to 0.69)	1 (− 0.47 to 0.49)
Dibenzo(a,h)anthracene—PAH	− 0.07	− 0.14	2 (− 0.21 to 0.55)	2 (− 0.89 to 0.59)	2 (− 0.49 to 0.54)	2 (− 0.89 to 0.58)
Hydrochloric acid	− 0.08	− 0.07	1 (− 0.01 to 0.49)	2 (− 0.32 to 0.61)	0 (− 0.50 to 0.39)	2 (− 0.71 to 0.49)
*High Density Livestock Operation	− 0.08	− 0.08	0 (− 0.41 to 0.14)	0 (− 0.39 to 0.13)	0 (− 0.41 to 0.14)	0 (− 0.18 to 0.14)
Polymeric diphenylmethane diisocyanate	− 0.09	− 0.10	1 (− 0.15 to 0.41)	1 (− 0.15 to 0.53)	1 (− 0.15 to 0.75)	0 (− 0.15 to 0.20)
7,12-Dimethylbenz(a)anthracene—PAH	− 0.10	− 0.25	1 (− 0.21 to 0.48)	1 (− 0.89 to 0.43)	1 (− 0.49 to 0.51)	1 (− 0.89 to 0.45)
3-Methylcholanthrene—PAH	− 0.10	− 0.25	1 (− 0.21 to 0.49)	1 (− 0.89 to 0.44)	1 (− 0.49 to 0.52)	1 (− 0.89 to 0.46)
*Power Line	− 0.10	− 0.04	1 (− 0.84 to 0.41)	3 (− 0.31 to 0.67)	0 (− 0.86 to 0.26)	2 (− 0.31 to 0.53)
1,1,2-Trichloroethane	− 0.10	− 0.08	0 (− 0.20 to − 0.20)	0 (− 0.20 to − 0.20)	0 (− 0.26 to − 0.01)	0 (− 0.29 to − 0.01)
HCFC-142b	− 0.10	− 0.08	0 (− 0.20 to − 0.20)	0 (− 0.20 to − 0.20)	0 (− 0.26 to − 0.01)	0 (− 0.29 to − 0.01)
1,1,2,2-Tetrachloroethane	− 0.10	− 0.08	0 (− 0.20 to − 0.20)	0 (− 0.20 to − 0.20)	0 (− 0.26 to − 0.01)	0 (− 0.29 to − 0.01)
Carbon tetrachloride	− 0.10	− 0.08	0 (− 0.20 to − 0.20)	0 (− 0.20 to − 0.20)	0 (− 0.26 to − 0.01)	0 (− 0.29 to − 0.01)
Pentachloroethane	− 0.10	− 0.08	0 (− 0.20 to − 0.20)	0 (− 0.20 to − 0.20)	0 (− 0.26 to − 0.01)	0 (− 0.29 to − 0.01)
Dicyclopentadiene	− 0.10	− 0.08	0 (− 0.20 to 0.00)	0 (− 0.20 to − 0.01)	0 (− 0.26 to − 0.01)	0 (− 0.29 to − 0.01)
1,3-Butadiene	− 0.10	− 0.08	0 (− 0.20 to 0.00)	0 (− 0.20 to − 0.01)	0 (− 0.26 to − 0.01)	0 (− 0.29 to − 0.01)
Chloroethane	− 0.10	− 0.08	0 (− 0.20 to − 0.20)	0 (− 0.20 to − 0.20)	0 (− 0.28 to − 0.01)	0 (− 0.31 to − 0.01)
Chloroform	− 0.10	− 0.08	0 (− 0.20 to − 0.20)	0 (− 0.20 to − 0.20)	0 (− 0.31 to − 0.01)	0 (− 0.31 to − 0.01)
Vinyl chloride	− 0.10	− 0.08	0 (− 0.20 to − 0.20)	0 (− 0.20 to − 0.20)	0 (− 0.32 to − 0.01)	0 (− 0.34 to − 0.01)
Zinc (and its compounds)	− 0.11	− 0.17	2 (− 0.19 to 0.54)	3 (− 0.86 to 0.58)	2 (− 0.50 to 0.54)	2 (− 0.86 to 0.59)
Arsenic (and its compounds)	− 0.11	− 0.13	1 (− 0.18 to 0.55)	1 (− 0.32 to 0.59)	1 (− 0.50 to 0.52)	1 (− 0.71 to 0.57)
Tetrachloroethylene	− 0.12	− 0.12	1 (0.42 to 0.42)	1 (0.47 to 0.47)	1 (− 0.24 to 0.41)	1 (− 0.27 to 0.46)
Dioxins and furans—total	− 0.12	− 0.12	2 (− 0.10 to 0.66)	1 (− 0.32 to 0.70)	2 (− 0.50 to 0.67)	1 (− 0.71 to 0.71)
Nitrogen oxides (expressed as NO_2_)	− 0.13	− 0.19	**6 (**− **0.26 to 0.90)**	2 (− 0.83 to 0.61)	3 (− 0.27 to 0.91)	1 (− 0.83 to 0.48)
Chlorine dioxide	− 0.13	− 0.15	1 (− 0.15 to 0.49)	1 (− 0.32 to 0.44)	1 (− 0.49 to 0.52)	1 (− 0.72 to 0.46)
Hexachlorobenzene	− 0.13	− 0.14	2 (− 0.03 to 0.49)	2 (− 0.32 to 0.48)	2 (− 0.50 to 0.52)	2 (− 0.71 to 0.52)
*Socioeconomic Index	− 0.14	− 0.14	0 (− 0.59 to 0.21)	0 (− 0.58 to 0.17)	0 (− 0.59 to 0.26)	0 (− 0.58 to 0.24)
1,2-Dichloroethane	− 0.15	− 0.08	0 (− 0.42 to − 0.20)	0 (− 0.20 to 0.12)	0 (− 0.26 to − 0.01)	0 (− 0.29 to − 0.01)
Acetonitrile	− 0.15	− 0.15	0 (0.13 to 0.13)	0 (0.18 to 0.18)	0 (0.08 to 0.15)	0 (0.14 to 0.15)
1,4-Dioxane	− 0.15	− 0.08	0 (− 0.42 to − 0.20)	0 (− 0.20 to 0.12)	0 (− 0.30 to − 0.30)	0 (− 0.32 to − 0.32)
HCFC-22	− 0.15	− 0.08	0 (− 0.42 to − 0.20)	0 (− 0.20 to 0.12)	0 (− 0.30 to − 0.01)	0 (− 0.32 to − 0.01)
*Water body	− 0.17	− 0.12	1 (− 0.62 to 0.49)	0 (− 0.55 to 0.14)	1 (− 0.70 to 0.49)	0 (− 0.63 to 0.22)
Phenol (and its salts)	− 0.17	− 0.18	1 (− 0.13 to 0.41)	1 (− 0.10 to 0.53)	1 (− 0.18 to 0.75)	0 (− 0.19 to 0.20)
Triethylamine	− 0.17	− 0.18	0 (0.06 to 0.06)	0 (0.11 to 0.11)	0 (0.02 to 0.15)	0 (0.07 to 0.15)
Acenaphthylene—PAH	− 0.18	− 0.26	1 (− 0.21 to 0.49)	1 (− 0.89 to 0.44)	1 (− 0.49 to 0.52)	1 (− 0.89 to 0.46)
Nitrilotriacetic acid (and its salts)	− 0.19	− 0.19	0 (− 0.35 to − 0.35)	0 (− 0.35 to − 0.35)	0 (− 0.40 to − 0.40)	0 (− 0.40 to − 0.40)
Mercury (and its compounds)	− 0.19	− 0.21	0 (− 0.18 to 0.37)	1 (− 0.33 to 0.42)	0 (− 0.50 to 0.23)	0 (− 0.71 to 0.28)
Nitrate ion in solution at pH > = 6.0	− 0.19	− 0.19	0 (− 0.33 to − 0.33)	0 (− 0.31 to − 0.31)	0 (− 0.38 to − 0.38)	0 (− 0.37 to − 0.37)
Nitric acid	− 0.19	− 0.19	0 (− 0.34 to − 0.34)	0 (− 0.34 to − 0.34)	0 (− 0.40 to − 0.40)	0 (− 0.39 to − 0.39)
Selenium (and its compounds)	− 0.19	− 0.21	1 (− 0.06 to 0.56)	1 (− 0.43 to 0.61)	1 (− 0.16 to 0.53)	1 (− 0.67 to 0.58)
Silver (and its compounds)	− 0.20	− 0.20	0 (− 0.36 to − 0.36)	0 (− 0.35 to − 0.35)	0 (− 0.42 to − 0.42)	0 (− 0.41 to − 0.41)
Antimony (and its compounds)	− 0.20	− 0.20	0 (− 0.37 to − 0.37)	0 (− 0.36 to − 0.36)	0 (− 0.42 to − 0.18)	0 (− 0.41 to − 0.13)
Cadmium (and its compounds)	− 0.20	− 0.21	3 (− 0.09 to 0.53)	2 (− 0.03 to 0.60)	1 (− 0.34 to 0.44)	2 (− 0.66 to 0.49)
Copper (and its compounds)	− 0.20	− 0.23	1 (− 0.18 to 0.62)	2 (− 0.34 to 0.65)	1 (− 0.21 to 0.61)	1 (− 0.35 to 0.64)
*Wildfire	− 0.24	− 0.28	1 (− 0.35 to 0.57)	0 (− 0.64 to 0.39)	3 (− 0.47 to 0.57)	1 (− 0.71 to 0.75)
Cobalt (and its compounds)	− 0.30	− 0.39	0 (− 0.43 to − 0.03)	1 (− 0.88 to 0.46)	0 (− 0.42 to − 0.11)	0 (− 0.88 to 0.00)
*Cultivated Land	− **0.47**	− **0.41**	0 (− 0.33 to 0.17)	1 (− 0.34 to 0.49)	1 (− 0.61 to 0.81)	2 (− 0.62 to 0.54)
*Oil/Gas Wellpad	− **0.53**	− **0.49**	0 (− 0.45 to 0.31)	0 (− 0.34 to 0.33)	2 (− 0.81 to 0.80)	2 (− 0.74 to 0.79)
*Vegetation	− **0.56**	− **0.48**	2 (− 0.50 to 0.80)	3 (− 0.52 to 0.48)	1 (− 0.48 to 0.83)	3 (− 0.52 to 0.58)

In the right half of the table, the count of units exceeding *rho* > 0.40 and the range are shown for the data aggregated by health regions and airshed zones. Variables having a rho > 0.4 for the province or for 4 or more sub-provincial units are indicated by bold font

The dilution effect of spreading the hazards across the large study area highlighted regional importance. Using the criteria of four or more health regions having a *rho* greater than 0.40 indicated the importance of Nitrogen oxides, Sulphur dioxide, Particulate Matter less than or equal to 2.5 μ (PM_2.5_), and Acetaldehyde with SGA. The same criteria identified Xylene, Mine Site, Manganese, and Lead for LBWT. Four or more airshed zones having a *rho* greater than 0.40 highlighted Sulphur dioxide and Acetaldehyde with SGA, and Xylene, Particulate Matter less than or equal to 10 microns (PM_10_), and PM_2.5_ for LBWT.

The number of unique environmental variables having *rho* values greater than 0.40 province-wide or within four or more sub-provincial units totaled 30 (24 air substances and 6 land-based).

### Spatial distribution of the indices

**Fig. 7:**
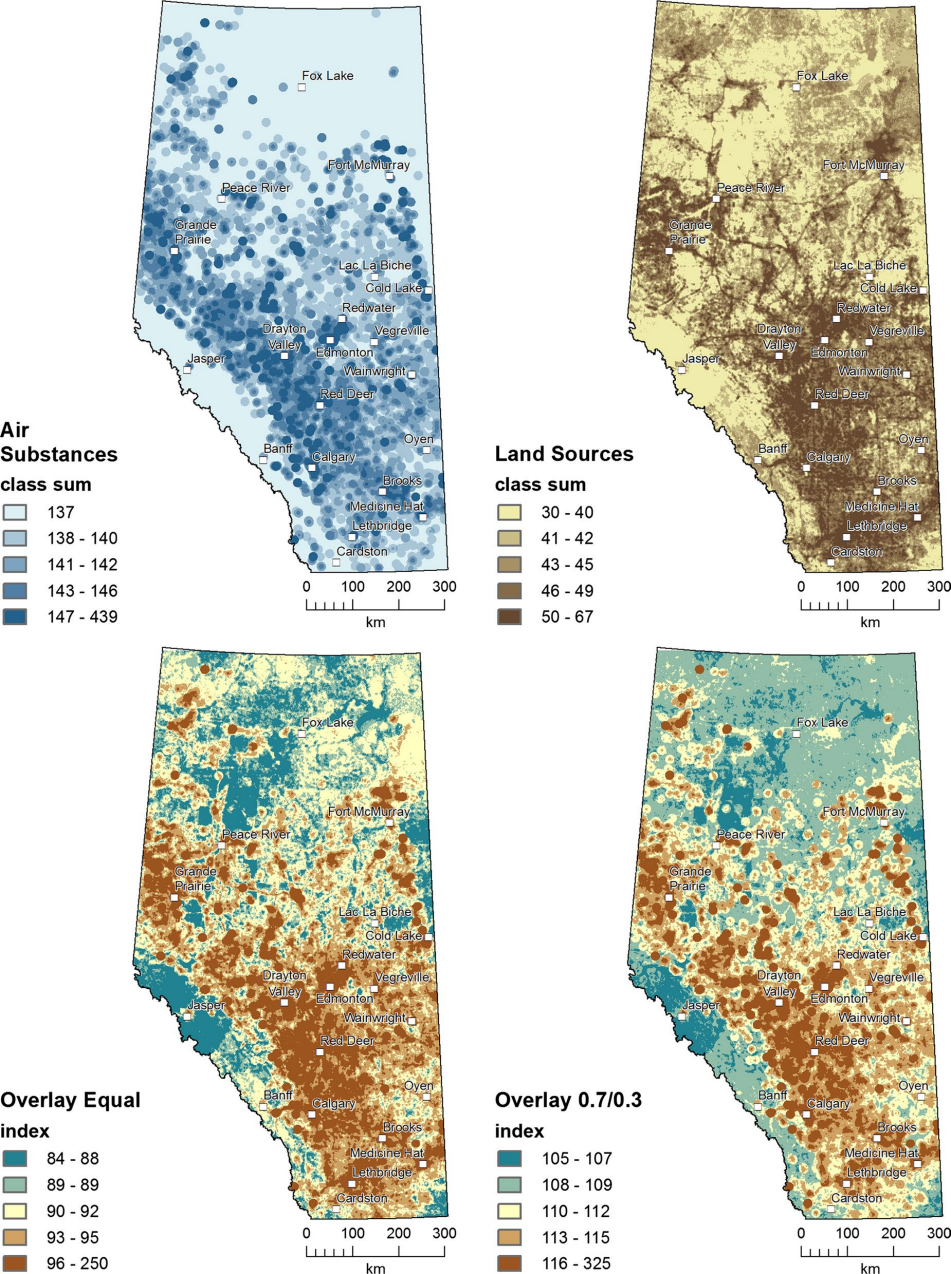
Weighted sum overlays for air substances and land-based sources were combined as equal and 0.7/0.3 weighted indices to identify the most hazardous locations

Figure 7 maps the results from the weighted overlay sum of the five class rankings for 136 emitted air substances, 18 land sources, the overlay equal weighting of both, and the Overlay 0.7/0.3 weighting of air substances and land. The distribution of the higher rankings spatially coincides with Alberta’s populated places, except for higher values along the foothills, the Fort McMurray oil sands area in the north, and some scattered areas in the northeast. Quantile class breaks were used to visualize the contrast of higher to lower areas.

### Associations with the hazards and indices

The actual index values were used for the correlations with ABO DKD (Table 4). The correlations of the overlay indices with ABOs were very low for the entire province. The Air Substances were highest for both SGA (*rho* = 0.21) and LBWT (*rho* = 0.16). Land Factor correlations were slightly negative for SGA (*rho* = − 0.26) and LBWT (*rho* = − 0.23). Both overlay indices were lower than the Air Substances for SGA: Overlay Equal had a *rho* = 0.18 and Overlay 0.7/0.3 had a *rho* = 0.15. Overlay Equal was lower for LBWT (*rho* = 0.13) but Overlay 0.7/0.3 was higher (*rho* = 0.20).

**Table 4:** Spearman’s rank correlations of small for gestational age (SGA) and low birth weight at term (LBWT) with air substances, land sources, and weighted sum overlay indices for the entire province of Alberta

Index name	Inputs	SGA *rho*	LBWT *rho*
Air substances	Sum of 136 variables classified to 5 quantiles	0.21	0.16
Land sources	Sum of 18 variables classified to 5 quantiles	− 0.26	− 0.23
Overlay equal	Air substances + land sources	0.18	0.13
Overlay 0.7/0.3	0.7 * air substances + 0.3 * land sources	0.15	0.20

**Fig. 8:**
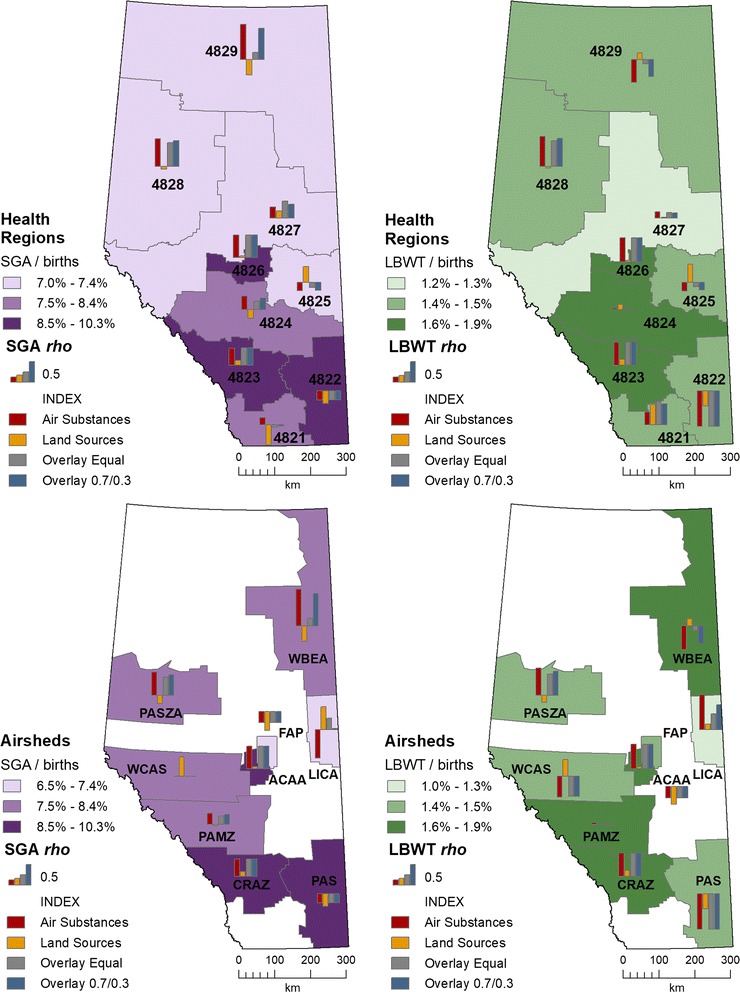
Spearman’s rank correlations of each adverse birth outcome with indices—shown as bar charts in each health region or airshed. Background maps show ratios

Figure 8 displays index correlations with ABOs, by health region and airshed zone. In the graph symbols, longer bars mean greater association and bar direction designates positive (up) or negative (down). The air substances and land-based sources were included to demonstrate how much of an effect each had on the indices. The following indices had correlations greater than 0.40 with an ABO:

- Air Substances with SGA in four health regions—4829 (*rho* = 0.85), 4828 (*rho* = 0.67), 4826 (*rho* = 0.55), 4823 (*rho* = 0.42); and with LBWT in three health regions—4828 (*rho* = 0.73), 4826 (*rho* = 0.59), and 4823 (*rho* = 0.56).
- Air Substances with SGA in four airshed zones—WBEA (*rho* = 0.89), PASZA (*rho* = 0.57), ACAA (*rho* = 0.55) and CRAZ (*rho* = 0.42); and with LBWT in four airshed zones—LICA (*rho* = 0.85), PASZA (*rho* = 0.66), ACAA (*rho* = 0.60), and CRAZ (*rho* = 0.56).
- Land sources were weakly associated with both SGA and LBWT in most health regions and airshed zones.
- Overlay Equal index with SGA in four health regions—4828 (*rho* = 0.58), 4826 (*rho* = 0.54), 4823 (*rho* = 0.42), and 4827 (*rho* = 0.42); and with LBWT in four health regions—4828 (*rho* = 0.0.63), 4826 (*rho* = 0.0.59), 4821 (*rho* = 0.57), and 4823 (*rho* = 0.57).
- Overlay Equal index with SGA in three airshed zones—ACAA (*rho* = 0.55), PASZA (*rho* = 0.45), and CRAZ (*rho* = 0.42); and with LBWT in three airshed zones—ACAA (*rho* = 0.60), CRAZ (*rho* = 0.57), and PASZA (*rho* = 0.51).
- Overlay 0.7/0.3 index with SGA in four health regions—4829 (*rho* = 0.75), 4828 (*rho* = 0.62), 4826 (*rho* = 0.55), and 4823 (*rho* = 0.42); and with LBWT in four health regions—4828 (*rho* = 0.68), 4826 (*rho* = 0.59), 4823 (*rho* = 0.57), and 4821 (*rho* = 0.51).
- Overlay 0.7/0.3 index with SGA in four airshed zones—WBEA (*rho* = 0.78), ACAA (*rho* = 0.55), PASZA (*rho* = 0.50), and CRAZ (*rho* = 0.42); and with LBWT in four airshed zones—LICA (*rho* = 0.60), ACAA (*rho* = 0.60), PASZA (*rho* = 0.59), and CRAZ (*rho* = 0.57).

The health regions having the least association with SGA were 4821, 4822, 4824, and 4825; with LBWT these were 4822, 4824, 4827, and 4829. The airshed zones having the least association with SGA were FAP, PAMZ, PAS, and WCAS; with LBWT these were FAP, PAMZ, PAS, and WBEA.

SGA and LBWT were negatively correlated with *all* indices in health region 4822 and two airshed zones (PAS, FAP). The negative association also occurred in health region 4825 and airshed zone WCAS, but a higher positive correlation occurred with the Land Sources.

Using the criteria of correlations higher than 0.40, the Overlay 0.7/0.3 index had the highest overall count of sub-provincial units—both ABOs represented by at least 4 health regions and 4 airshed zones.

## Discussion

### Individual hazards

Of 136 NPRI substances reported in Alberta, 24 air-emitted substances had moderate correlations with one or both ABO DKD ratios. Of these, 2-Ethoxyethanol and Lead are recognized developmental toxicants [53, 54]. Acetaldehyde, Aluminum, Ethylene oxide, Isopropyl alcohol, Nickel, Nitrogen oxides, PM_10_, PM_2.5_, Sulphur dioxide, Xylene, Chromium, Hydrogen sulphide, Manganese, Phosphorus, and Quinoline are suspected developmental toxicants, with more than half of the air substances associated with decreased fetal/offspring weight in animal studies [53, 54]. The following air substances are neither recognized or suspected as no studies were reported: Aniline, Asbestos, Cyclohexane, i-Butyl alcohol, Toluene-2,4-diisocyanate, Toluene-2,6-diisocyanate, and Toluenediisocyanate (note: the latter three have been combined in later versions of the NPRI database [9]).

Of the 18 land sources mapped, 6 had moderate correlations with one or both ABOs. Provincially, Cultivated Land was negatively associated with SGA and LBW (likely because residences were not inside agricultural fields), but some regions were positive, similar to the Almberg et al. [55] study on proximity to pesticide-treated agricultural fields. Proximity to Mine Sites were associated for 2–3 health regions or airsheds; a related study found positive association for a single mine site indicating this is likely a more localized factor [56]. Nighttime Lights have not been explored with ABOs; however, breast cancer, which has other similar exposures, has a positive association [44, 57]. The smaller area airsheds showed high correlations of ABOs with Oil/Gas Wellpads, but was negative for the entire province and by health regions; mixed associations were also reported by Mckenzie et al. [58] and Casey et al. [59]. The moderate to higher correlations of Roads match much published research on the effect of maternal proximity to roads [60, 61]. Green or natural Vegetation was negatively correlated at the provincial level, but very mixed within health regions and airsheds; the sub-provincial dissimilarity with other studies [62, 63] was likely affected by the radii, resolution of the satellite sources, and the widely varying ecoregions in the province.

### Ambient hazard indices

Both indices identified where there was an accumulation of hazards and therefore directly addressed the hypothesis that there were more small newborns where there were more outdoor hazards during the mothers’ pregnancies. Since we were interested in preserving combined effects that the industrial air substances contributed to the outdoor environment, we weighted the sum of those more highly than the sum of all the land-based sources. Province-wide, the Overlay Equal index better identified SGA and the Overlay 0.7/0.3 better identified LBWT.

Differences in index associations were likely due to the spatial distributions (i.e. DKD) of the ABOs. Both SGA and LBWT showed that hot spots did not occur strictly within the large urban centers. Calgary and Edmonton exhibited higher ratio classes, but not for their entire core. The peripheral edges of the Calgary-Red Deer corridor, the communities along the Banff-Calgary-Brooks corridor, the Fort McMurray surroundings, and the northern Fox Creek area were high for both SGA and LBWT. Jasper and south-east Alberta had higher SGA, while the communities west and east of Edmonton had higher LBWT. The distributions of the type of ABO spatially varied across the province—differences that may have been due in part to population and behavior, but also visually collocated with the higher amounts of outdoor hazard mapping.

Separately, the air substances and land sources varied in association with the ABO distributions. On the provincial scale, there were 13 hazards spatially related to both the SGA and LBWT ratios. Assessing the relationships sub-provincially found many more factors involved, including those already supported in the scientific literature, including: nitrogen oxides, particulate matter (PM_2.5_ and PM_10_), and sulphur dioxide.

Despite the disparate boundaries, spatially corresponding health regions (HR) and airshed zones (AZ) had comparable patterns in spatial relationships to the hazard indices. HR 4822/AZ PAS had highly negative correlations with all indices, suggesting that factors other than the outdoor environment may be more important in these regions. HR 4829/AZ WBEA exhibited opposite correlations with indices: SGA was positive and LBWT was negative. HR 4826/AZ ACAA and FAP and HR 4823/AS CRAZ for SGA and LBWT were positively correlated with the indices—these are the more populated regions. 4828/PASZA also had positive index correlations with SGA and LBWT. HR 4824/AZ PAMZ for SGA was positive with the indices, but for LBWT had no association. The reverse was found in HR 4821 (no corresponding AZ), where SGA was negative and LBWT was positive. HR 4825 had no relationship with the ABOs, and AZ LICA had no association with SGA and a positive one with LBWT. HR 4827 and AZ WCAS are too large and diverse to compare. Inconsistent relationships for each ABO with the indices may be due to: (1) the variable geography within the administrative boundaries; (2) differences in etiology of the ABOs; and/or (2) the actual distribution of each ABO exhibiting slightly different patterns: SGA and LBWT appear to be more of a heartland issue.

The combination of the outdoor hazards into a single index were very weakly associated with SGA and LBWT provincially. This was not surprising given Alberta includes forestry, agriculture, and energy extraction activities, thus yielding diverse “pockets” of different pollutants. Analyzing smaller geographic areas, based on health regions or airsheds, helped recognize possible differences in the outdoor environmental factors.

The large area of some units may capture populations that are more similar in size to the smaller units, but the environmental variability may have diluted the effects of hazards. The sub-provincial units that had negative correlations will need further analysis to determine the regionally important hazards. Relationships found here show that province-wide (i.e. large region) approaches to outdoor hazards may be inappropriate or inefficient. Where health regions and airshed zones are more similar, policy and monitoring may be more agreeable.

Existing ambient hazard indices are not available for comparison. Environmental Quality Indices (EQI), such as those developed by Messer et al. [28] and Stieb et al. [64] depict the state-of-the-environment from actual measured conditions [27]. The Air Quality Health Index (AQHI) by Stieb et al. does a very good job at aggregating the monitored criteria air contaminants for risk communication. Messer’s EQI was associated with pre-term birth [65], but still has the limitation of fixed administrative units. And because a main goal was a continuous index, we were unable to incorporate an effective rural classification without the introduction of administrative boundaries, as done by Messer et al. Our more ecologically-encompassing index incorporated industrial air pollutants and land-based sources, similar to the holistic model developed for a single urban area by Tarocco et al. [66].

## Limitations

We analyzed the entire registered birth population for the study period that had valid locations. The 6-character postal codes provided good accuracy for urban neighborhoods, especially within the context of the 250-m cell size, but the rural residences were not as exact. DMTI Spatial had applied algorithms to weight the postal code local delivery area centroid toward the more populated communities [41], but that did not guarantee an actual residence contained within the cell. The problem of rural resolution was exhibited by oil–gas wellpads and agricultural land that may be closer to actual residences, but postal codes were not accurate enough due to too large of delivery areas for the centroids.

Although there is concern that the mother did not live at that postal code for the entire pregnancy, previous research determined low mobility during pregnancy and any relatively short distances moved did not substantially change the exposure assignment [67].

The spatial data for the independent variables were restricted to publicly available sources that may not have had the most temporally appropriate capture date of the mapped features. We also did not have access to reliable province-wide data for other possible environmental factors, such as water quality, noise, or non-industrial pollution sources. And as suitable as the NPRI data were, the values were annually reported estimates and not actual measurements [9]. Despite these shortcomings, the available data provided an as inclusive as possible foundation for the index.

Many of the GIS methods involved the selection of radius distances. The size of the radius used in calculating the DKD affected how “hot” an area appeared, and may have exaggerated the extent for large distances; the 25-km radius may have been too large for rural communities with diverse topographies. When estimating air-emitted pollutants, wind would have varied by season and throughout the years; therefore, the use of circular shapes in calculating the tonnes per area may not have accurately reflected wind-dispersion for some areas. The conservative 10 km radius for spreading the air substances may have remedied this for upwind locations, but potentially underrepresented it for downwind locations. For the index, not all variables may be equally important, but the use of expert judgment would have introduced subjectivity that was not reproducible. Therefore, the equal treatment of the air substances and land sources in the overlay analyses was used.

The correlation threshold value of 0.40 may have overrepresented the inclusion of some of the independent variables. The choice of this statistical threshold was based on inspection of the data to ensure that a wide variety of hazards would be represented and not erroneously overlooked due to the modifiable areal unit problem introduced by the boundaries of the sub-provincial units [68].

It is important to stress that our research was not able to find causal relationships, but identified where outdoor environmental hazards collocate with residences of mothers who gave birth to abnormally small newborns.

### Strengths

The calculations of the outdoor environmental variables were continuous and covered the entire study area. Therefore, the DKD calculation of the SGA and LBWT ratios was appropriately consistent because it also was not confined to arbitrary geographical boundaries. Aggregation early in the analysis would have produced an inflexible distribution of the ABOs. The introduction of health regions and airsheds afterward allowed for scenario investigations relevant to health care administration, policy implications, and airshed monitoring.

The primary outdoor pollutants associated with abnormally small newborns agreed with published research, but additional unstudied air substances were discovered. For many regions, the reduction of data into a single index was achievable.

The development and application of the ambient health hazard indices for any study area, any time period, and where relevant data are available is simplified by the reverse suitability approach in a standard GIS. The distance-centered methods and weighted sum overlay, commonly used in wildlife habitat studies, are also relevant to human habitat related to various environmental health outcomes.

## Conclusion

This is to date the first study on abnormally small newborns that used a combination of multiple outdoor variables over a large geographic area. Our results showed that SGA and LBWT varied sub-provincially with outdoor environmental factors, suggesting that provincial government should be aware of multiple sources of place-dependent exposures. Summing up class rankings of hazards provided a simple model for correlating with the sub-provincial distributions of ABO. There were regions/airsheds that were higher than the national and provincial rates. The temporal nuances had been masked by combining all years: spatial patterns in the hazards and birth outcomes likely varied through time; therefore, future research should consider the timing of exposures. Research should also combine the vertices of habitat, population, and behavior to investigate the complex interactions of the outdoor hazards found here by including maternal characteristics revealed in traditional epidemiological studies. We found that the industrial air substances were important—and the Overlay 0.7/0.3 weighted index had the most associations in the sub-provincial units. Therefore, both the individual air substance associations and the convenient single-measure index provide complementary information to move us toward a better understanding of the links between the outdoor environment and birthweight. Mapping the outdoor environmental hazards for mothers giving birth to abnormally small newborns provides insight for preventative or remedial recommendations *where* they may be needed to help determine healthier futures.

## Abbreviations

ABO: adverse birth outcome
APHP: Alberta perinatal health program
AZ: airshed zone
DoMiNO: data mining and neonatal outcomes
DKD: double kernel density
GIS: geographical information systems
HR: health region
LBWT: low birth weight at term
NPRI: national pollutant release inventory
SGA: small for gestational age
